# Altered structural connectivity in olfactory disfunction after mild COVID-19 using probabilistic tractography

**DOI:** 10.1038/s41598-023-40115-7

**Published:** 2023-08-09

**Authors:** Diógenes Diego de Carvalho Bispo, Pedro Renato de Paula Brandão, Danilo Assis Pereira, Fernando Bisinoto Maluf, Bruna Arrais Dias, Hugo Rafael Paranhos, Felipe von Glehn, Augusto César Penalva de Oliveira, Alexandre Anderson de Sousa Munhoz Soares, Maxime Descoteaux, Neysa Aparecida Tinoco Regattieri

**Affiliations:** 1 Diagnostic Imaging Unit, Brasilia University Hospital, University of Brasilia, Darcy Ribeiro Campus, Asa Norte, Brasilia, Distrito Federal Brazil; 2 Faculty of Medicine, University of Brasilia, Brasilia, Distrito Federal Brazil; 3 Department of Radiology, Hospital Santa Marta, Taguatinga, Distrito Federal Brazil; 4 Neuroscience and Behavior Lab, University of Brasilia, Brasilia, Distrito Federal Brazil; 5 Hospital Sírio-Libanês, Brasilia, Distrito Federal Brazil; 6 Advanced Psychometry Laboratory, Brazilian Institute of Neuropsychology and Cognitive Sciences, Brasilia, Distrito Federal Brazil; 7 Department of Neurology, Instituto de Infectologia Emílio Ribas, São Paulo, Brazil; 8 Sherbrooke Connectivity Imaging Lab, University of Sherbrooke, Sherbrooke, QC Canada; 9 Imeka Solutions Inc, Sherbrooke, QC Canada

**Keywords:** Olfactory system, Neural circuits, Central nervous system infections, Image processing

## Abstract

We aimed to investigate changes in olfactory bulb volume and brain network in the white matter (WM) in patients with persistent olfactory disfunction (OD) following COVID-19. A cross-sectional study evaluated 38 participants with OD after mild COVID-19 and 24 controls, including Sniffin' Sticks identification test (SS-16), MoCA, and brain magnetic resonance imaging. Network-Based Statistics (NBS) and graph theoretical analysis were used to explore the WM. The COVID-19 group had reduced olfactory bulb volume compared to controls. In NBS, COVID-19 patients showed increased structural connectivity in a subnetwork comprising parietal brain regions. Regarding global network topological properties, patients exhibited lower global and local efficiency and higher assortativity than controls. Concerning local network topological properties, patients had reduced local efficiency (left lateral orbital gyrus and pallidum), increased clustering (left lateral orbital gyrus), increased nodal strength (right anterior orbital gyrus), and reduced nodal strength (left amygdala). SS-16 test score was negatively correlated with clustering of whole-brain WM in the COVID-19 group. Thus, patients with OD after COVID-19 had relevant WM network dysfunction with increased connectivity in the parietal sensory cortex. Reduced integration and increased segregation are observed within olfactory-related brain areas might be due to compensatory plasticity mechanisms devoted to recovering olfactory function.

## Introduction

Since its discovery in Wuhan, China, in late 2019, coronavirus disease 2019 (COVID-19), caused by severe acute respiratory syndrome coronavirus 2 (SARS-CoV-2), has resulted in more than 600 million infected and 6 million deaths^1^ and put unprecedented pressure on social, economic and health systems around the world^2^. While initial research on COVID-19 has focused on acute illnesses, it lately has become clear that long-term consequences occur^3^. Many survivors of acute infection have persistent and disabling neurological symptoms, which can have socioeconomic and personal consequences. It is, therefore, imperative that there is a thorough understanding of evolving clinical syndromes and underlying pathophysiological mechanisms, allowing rational therapeutic interventions to be implemented quickly^2^.

Olfactory dysfunction has variable severity, including anosmia, hyposmia, and parosmia, and affects 30–70% of patients with COVID-19^4^. It occurs early in the course of infection, with no direct association with disease severity or viral burden^5^. In one study, hyposmia was the first clinically presenting symptom in around 12% of patients^6^. In most cases, recovery is spontaneous within 3 to 4 weeks^5,7^. However, some patients develop persistent olfactory impairment up to 12 months after infection, suggesting that the damage to the olfactory system may be severe or permanent^5^.

Several hypotheses were proposed to explain the underlying mechanism for olfactory dysfunction in COVID-19^8,9^. The most notable theory regarding COVID-related hyposmia is the direct infection of olfactory receptor neurons by SARS-CoV-2 through the nasal mucosa. However, there is conflicting evidence on whether SARS-CoV-2 can indeed infect these neurons^10^. Angiotensin-converting enzyme 2 receptors—the target molecules for SARS-CoV-2, are not expressed in neural cells but by non-neuronal support cells in the olfactory epithelium. The lack of direct neuronal damage could justify the rapid recovery of olfactory function in most patients^11^. Despite this, SARS-CoV-2 infection seems to generate axonal pathology and microvasculopathy in the olfactory bulbs and tracts in those with olfactory alterations, due to local inflammation^12^.

Olfactory dysfunction during or after COVID-19 represents a marker of neurological disease and can be assessed with olfactory nerve imaging. Magnetic resonance imaging (MRI) can help evaluate patients with anosmia and hyposmia because it allows for elaborate visualization and measurement of the olfactory anatomical structures. Yet, studies that describe MRI-based anatomical changes in olfactory structures in COVID-19 are sparse and mainly represented by case reports. Despite this, a reduction in olfactory bulbs was described in 36 participants who had COVID-19 olfactory dysfunction compared to a control group 2 to 8 weeks after infection^13^, and in 196 subjects who had COVID-19 compared to controls 1 to 582 days after disease onset^14^. The association between olfactory bulb atrophy and severity of the olfactory dysfunction was not evaluated in these studies. Furthermore, the impact on brain connections is relatively unknown, especially in sensory and olfactory-related regions.

Diffusion-weighted magnetic resonance imaging (dMRI)-derived tractography is an advanced technique that may be used to investigate the mechanisms underlying anosmia by reconstructing major brain fiber pathways. This method allows for the mapping of white matter (WM) pathways through voxel-wise fiber orientations. It enables the reconstruction of structural connectivity matrices, generating networks that represent parts or the whole brain's anatomical organization, with streamlines serving as proxies for WM fiber bundles^15^. The Convex Optimization Modeling for Microstructure Informed Tractography 2 (COMMIT2) framework is used to remove false positive brain connections by assigning to each streamline its contribution to the dMRI signal, while imposing an anatomical regularization encouraging streamlines to group together as bundles in the connectivity matrix^16,17^. This filtering method significantly improves the accuracy of the resulting structural connectomes, thereby enhancing the reliability of the findings^18^.

Graph analysis is used to explore changes in the WM network based on graph theory^18^. After defining the nodes and edges (connections between regions), graph theory metrics represent distinct aspects of global or local network connectivity. A small-world architecture, for instance, indicates that the minimum path length between any pair of nodes is approximately equivalent to a comparable random network, but the network nodes have greater local interconnectivity or cliquishness than a random network^15^. The relationship between olfaction and brain network metrics is intimate. Studies have shown that hyposmia in aging and neurodegeneration relates to WM disconnection using graph analysis methods^19,20^, whereas individuals with the highest olfactory abilities, such as *sommeliers*, exhibit increased functional network connectivity and higher small-world topology than controls^21^. However, there is still a need for further exploration of WM network changes in non-neurodegenerative hyposmia. The use of graph theory in understanding changes in the WM network may help elucidate the underlying mechanisms of olfactory dysfunction.

The current study used a cross-sectional design to examine changes in the olfactory bulb volume and investigate brain networks in patients after COVID-19 compared to a control group. Our secondary objective was to determine whether there was an association between olfactory bulb volume, structural connectivity measures, and olfactory performance.

## Results

### Demographic and clinical characteristics

In this study, we recruited a total of 67 individuals, out of which three participants from the COVID-19 group (COV +) were excluded. Two of the exclusions were due to MRI contraindications, while the third exclusion was due to a Montreal Cognitive Assessment (MoCA) score of less than 15. In addition, two participants from the control group (COV−) were excluded from the study. One exclusion was due to a positive SARS-CoV-2 IgG test result, while the other exclusion was due to the detection of a brain structural change on MRI. These exclusions were necessary to ensure the integrity and validity of the data obtained from the study population.

Clinical examinations, cognitive tests, and MRI were administered to a total of 62 participants, comprising 38 in the COV + group and 24 in the COV− group. The groups did not exhibit any significant differences in age (p = 0.520), sex (p = 0.550), education (p = 0.555), or comorbidity profiles (Table 1). The average time between COVID-19 diagnosis and study´s clinical/imaging procedures was 91.7 (± 26.0) days, with a range of 31 to 167 days. No subjects in the COV + group required hospitalization during the acute phase or thereafter.

**Table 1:** Demographic and clinical features (COV + and COV − groups).

Demographic and clinical characteristics	COVID-19 (COV +)	Control (COV−)	Statistic
	(n = 38, 61%)	(n = 24, 39%)	
Age	36.4 ± 9.5 (20, 56)	39.3 ± 12.9 (22, 60)	U = 411; p = 0.520^1^
Sex			
Male, n (%)	10 (26.3%)	8 (33.3%)	χ^2^ = 0.35; p = 0.550^2^
Female, n (%)	28 (73.7%)	16 (66.7%)	
Years of formal education	15.1 ± 3.2 (11, 24)	15.5 ± 3.0 (11, 20)	U = 415; p = 0.555^1^
Comorbidities, n (%)			
Hypertension	3 (7.9%)	0 (0.0%)	χ^2^ = 0.199; p = 0.160^2^
Diabetes mellitus	2 (5.3%)	1 (4.2%)	χ^2^ = 0.04; p = 0.840^2^
Obesity	1 (2.6%)	1 (4.2%)	χ^2^ = 0.11; p = 0.740^2^
Asthma/COPD	2 (5.3%)	2 (8.3%)	χ^2^ = 0.23; p = 0.630^2^
Allergic rhinosinusitis	11 (28.9%)	8 (33.3%)	χ^2^ = 0.13; p = 0.720^2^
Thyroid disorder	2 (5.3%)	1 (4.2%)	χ^2^ = 0.04; p = 0.840^2^
Mood disorder	4 (10.5%)	2 (8.3%)	χ^2^ = 0.08; p = 0.780^2^
Migraine	12 (31.6%)	7 (29.2%)	χ^2^ = 0.04; p = 0.840^2^
Sniffin’ Sticks identification test (SS-16)	11.4 ± 2.1 (6, 15)	13.6 ± 1.1 (12, 16)	U = 177; p < 0.001^1^
Montreal Cognitive Assessment (MoCA)	24.9 ± 3.2 (17, 30)	25.2 ± 3.4 (17, 30)	U = 426; p = 0.663^1^

*COPD* chronic obstructive pulmonary disease.

Data are shown as mean ± standard deviation (minimum, maximum) or n (%).

^1^Mann–Whitney U test.

^2^Chi-square test.

There was a significant difference between the two groups in the Sniffin' Sticks smell identification test (SS-16) score (p < 0.001). In the COV + group, 50% of patients were hyposmic (SS-16 test score below 12)^22^. None of the subjects in the COV− group had hyposmia. MoCA scores did not differ between groups (p = 0.663) (Table 1).

### Assessment of the olfactory bulbs

The olfactory bulbs of 53 participants (33 in the COV + group and 20 in the COV− group) were manually segmented. Nine participants were excluded due to movement or magnetic susceptibility artifacts, usually due to metallic material in the oral cavity. In the assessment of interobserver agreement for manual segmentation of the olfactory bulbs, Pearson's correlation coefficients were r = 0.877 for the right olfactory bulb and r = 0.900 for the left olfactory bulb (two-tailed, p < 0.001). The mean and standard deviation of the Dice similarity coefficient (DSC) for right and left olfactory bulbs were 0.815 + /− 0.0354 and 0.794 + /− 0.0483, respectively, indicating a good agreement between examiners.

**Figure 1:**
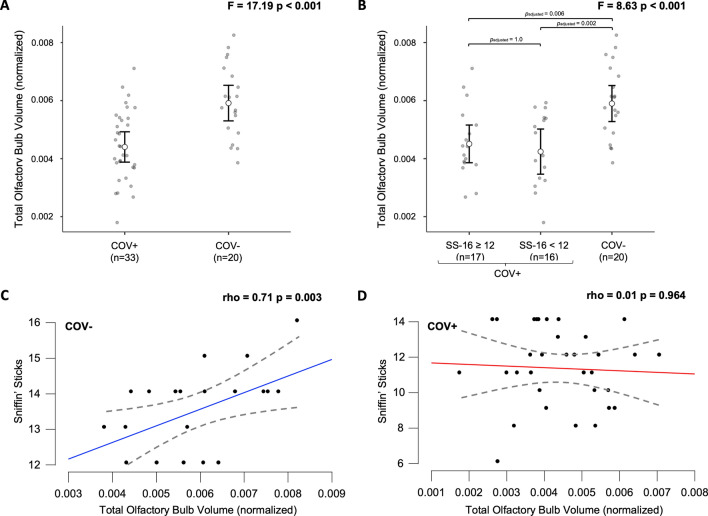
(**A**) Comparison of the normalized total olfactory bulb volume between the COV− and COV + groups using ANCOVA. (**B**) Comparison of the normalized total olfactory bulb volume among the COV-, COV + with SS-16 ≥ 12 and COV + with SS-16 < 12 subgroups using ANCOVA. (**C**) Association between normalized total olfactory bulb volume and SS-16 test score in the COV− group. (**D**) Association between normalized total olfactory bulb volume and SS-16 test score in the COV + group. *SS-16* Sniffin' Sticks smell identification test, *ANCOVA* analysis of covariance. *ANCOVA with covariates including sex, age, and allergic rhinosinusitis.

The volume of the right (t = − 4.19, p < 0.001), left (t = − 4.42, p < 0.001), and both (t = − 4.42, p < 0.001) olfactory bulbs was significantly reduced in the COV + group when compared to the COV− group (Table 2). This reduction in the normalized total olfactory bulb volume remained even after controlling for the sex, age, and allergic rhinosinusitis (F = 17.19, p < 0.001) (Fig. 1A).In the COV + group, there was no difference in normalized total olfactory bulb volume between participants with SS-16 < 12 and SS-16 ≥ 12 (p = 1.000, Bonferroni correction). The normalized total olfactory bulb volume was smaller in these COV + subgroups compared to the COV− group (Fig. 1B).

**Table 2:** Comparison of the olfactory bulb volume between COV + and COV − groups.

Measure	COVID-19 (COV +)	Control (COV−)	Statistic^1^	Adj p^2^
	(n = 33, 62%)	(n = 20, 38%)		
Right olfactory bulb volume (mm^3^)	32.2 (9.5)	43.2 (8.8)	t = − 4.19; p < 0.001	p < 0.001
Left olfactory bulb volume (mm^3^)	31.0 (9.4)	43.1 (10.2)	t = − 4.19; p < 0.001	p < 0.001
Total olfactory bulb volume (mm^3^)	63.2 (18.4)	86.3 (18.4)	t = − 4.42; p < 0.001	p < 0.001

All data are shown as mean (standard deviation).

^1^Independent sample t-test.

^2^Adjusted p value for FDR.

A positive correlation was found between total olfactory bulb volume and SS-16 test performance in the study sample (n = 53) (rho = 0.281, p = 0.014). In the control group, there was a positive association between the volume of the olfactory bulbs and the SS-16 test score (rho = 0.706, p = 0.003) (Fig. 1C). In the COV + group, there was no association between the volume of the olfactory bulbs and the total score of the SS-16 test score (rho = 0.009, p = 0.964) (Fig. 1D).

### Voxel-based diffusion imaging analysis

No between-group differences were observed for fractional anisotropy (FA), mean diffusivity (MD), radial diffusivity (RD), and axial diffusivity (AD) using Tract-based Spatial Statistics (TBSS), controlling for age and sex (p > 0.05).

### Network‐based statistics (NBS)

**Figure 2:**
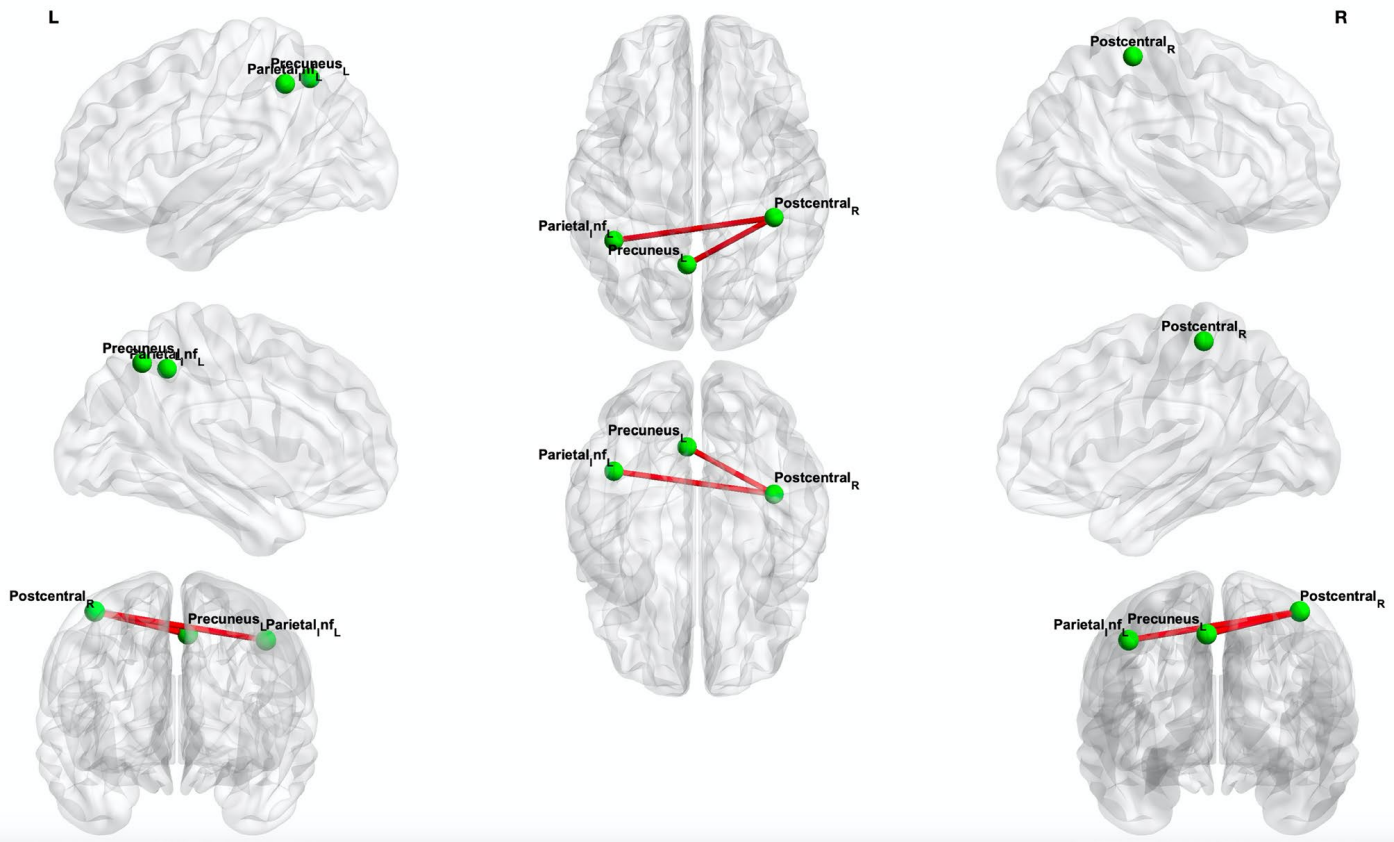
Whole-brain network-based statistics results. The connectivity analysis with threshold value t = 3.0 showed a subnetwork with greater connectivity in the COV + group compared to the COV− group (red edges). Significance was thresholded at p < 0.05. Permutations = 5000. *Inf* inferior, *R* right, *L* left.

Using a whole-brain exploratory analysis, NBS identified significant differences in structural connectivity between the COV + and COV− groups. Compared to the control group, COVID-19 patients exhibited significantly higher structural connectivity in a subnetwork composed of three brain regions (one on the right side and two on the left side) and two interhemispheric connections (threshold value t = 3.0, p < 0.05) (Fig. 2, Supplementary Table S1).

### Graph theory analysis: global network

COV + group showed lower global efficiency (p = 0.019) and local efficiency (p = 0.047) and higher assortativity (p = 0.027) than COV− group. No significant differences were found in other global network metrics (p > 0.05) (Table 3). Both groups had sigma > 1, which means they satisfied the criteria of a small-world (Table 3). In the COV + group, there was an association between the clustering and the SS-16 test score (rho =− 0.457, p = 0.019), controlling for sex, age, education, comorbidities, and time between COVID-19 diagnosis and study clinical/imaging procedures. No association was identified between global network measures and the SS-16 test score in the COV− group.

**Table 3:** Comparison of global network metrics between the COV + and COV −.

Measure	COVID-19 (COV+)	Control (COV-)	Statistic	Cohen's d
	(n = 32, 59%)	(n = 22, 41%)		
Betweenness centrality	0.00760 (0.00027)	0.00765 (0.00023)	t = − 0.695; p = 0.490^1^	–
Modularity	0.588 (0.577, 0.591)	0.585 (0.580, 0.590)	U = 327; p = 0.669^2^	–
Assortativity	0.01751 (0.0181)	0.00696 (0.0147)	t = 2.271; p = 0.027^1^*	0.629
Participation	0.248 (0.0111)	0.247 (0.0115)	t = 0.532; p = 0.597^1^	–
Clustering	1.18 (1.14, 1.25)	1.14 (1.09, 1.19)	U = 263; p = 0.120^1^	–
Nodal strength	91.1 (86.5, 92.0)	89.7 (88.4, 91.3)	U = 329; p = 0.694^2^	–
Local efficiency	49.8 (2.36)	51.2 (2.86)	t = − 2.031; p = 0.047^1^*	− 0.562
Global efficiency	39.1 (1.96)	40.6 (2.55)	t = − 2.413; p = 0.019^1^*	− 0.668
Density	0.147 (0.006)	0.150 (0.007)	t = − 1.808; p = 0.076^1^	–
Path length	97.4 (3.77)	98.7 (5.23)	t = − 1.046; p = 0.300^1^	–
Edge count	2.40 (0.0422)	2.40 (0.0421)	t = − 0.268; p = 0.790^1^	–
Omega	− 0.0266 (0.0244)	− 0.0205 (0.0202)	t = − 0.966; p = 0.339^1^	–
Sigma	1.47 (0.0491)	1.45 (0.0703)	t = 1.281; p = 0.206^1^	–

Modularity, clustering, and nodal strength are shown as median (interquartile range).

The other variables are shown as mean (standard deviation).

^1^Independent sample t-test.

^2^Mann-Whitney U test.

*p < 0.05.

### Graph theory analysis: local network

Compared with controls, patients exhibited reduced local efficiency (left lateral orbital gyrus and pallidum), increased clustering (left lateral orbital gyrus), increased nodal strength (right anterior orbital gyrus), and reduced nodal strength (left amygdala) after adjusting for multiple comparisons (Table 4, Supplementary Fig. S1).

**Table 4:** Comparison of local network metrics between the control and COVID-19 groups, adjusting for multiple comparisons (FDR).

Tract	N	Right Hemisphere			Left Hemisphere		
		statistic	p-value	adj p	statistic	p-value	adj p
Betweenness centrality							
Lateral orbital gyrus	54	0.482	0.632	ns	− 2.314	0.025	ns
Insula	54	− 0.826	0.412	ns	− 2.100	0.041	ns
Anterior cingulate cortex (supra)	54	0.916	0.364	ns	− 2.044	0.046	ns
Clustering							
Lateral orbital gyrus	54	− 0.102	0.919	ns	3.344	0.002	**0.006**
Insula	54	2.213	0.031	ns	− 0.518	0.607	ns
Edge count							
Anterior cingulate cortex (pre)	54	2.061	0.044	ns	− 2.576	0.013	**0.049**
Local efficiency							
Lateral orbital gyrus	54	− 0.664	0.510	ns	− 2.824	0.007	**0.025**
Hippocampus	54	− 1.024	0.311	ns	− 2.139	0.037	ns
Putamen	54	− 1.287	0.204	ns	− 2.063	0.044	ns
Pallidum	54	− 1.398	0.168	ns	− 2.532	0.014	**0.049**
Anterior cingulate cortex (supra)	54	− 1.861	0.069	ns	− 2.187	0.033	ns
Nodal strength							
Anterior orbital gyrus	54	2.477	0.017	**0.037**	1.781	0.081	ns
Lateral orbital gyrus	54	− 0.784	0.436	ns	2.070	0.044	ns
Amygdala	54	0.555	0.581	ns	− 2.703	0.009	**0.028**
Anterior cingulate cortex (sub)	54	0.713	0.479	ns	2.096	0.041	ns
Nucleus accumbens	54	− 1.639	0.107	ns	− 2.106	0.040	ns
Path length							
Anterior cingulate cortex (pre)	54	− 0.182	0.856	ns	− 2.241	0.029	ns
Participation							
Insula	54	0.536	0.594	ns	− 2.507	0.015	ns

Age and sex were included as covariates.

*Supra* supracallosal, *sub* subgenual, *pre* pregenual, *ns* not significant, *adj* adjusted.

Significant p values < 0.05 are indicated in bold font.

## Discussion

Our study findings reveal that patients with persistent subjective hyposmia following COVID-19 infection exhibited a 25% reduction in olfactory bulb volume at a mean follow-up of three months. In comparison to controls, SARS-CoV-2 infected hyposmic subjects demonstrated significant aberrations in the WM network, but no changes TBSS. The NBS analysis identified a subnetwork in the parietal sensory areas with increased connectivity. The global and local network topological properties demonstrated reduced integration and increased segregation, including olfactory-related brain areas (pallidum, amygdala, and orbitofrontal gyrus). Moreover, the SS-16 test score was negatively correlated with clustering in the COVID-19 group. Therefore, we hypothesize that changes in connectivity in the parietal sensory regions and olfaction-related brain areas may be due to compensatory plasticity mechanisms aimed at restoring olfactory function.

Unlike other upper respiratory infections, COVID-19 olfactory dysfunction is not associated with nasal discharge and conductive obstruction of the olfactory cleft, suggesting a neurological origin^23^. SARS-CoV-2 does not infect the sensory neurons, and sustentacular cells are the primary target of this virus in the olfactory mucosa^10,24–27^. Several studies have also reported changes in olfactory bulb volume, olfactory cleft volume, olfactory sulcus depth, and olfactory nerve morphology^28^. In our study, the COVID-19 olfactory bulb volume was significantly smaller than that of the control group, even after accounting for head size.

In the control group, olfactory bulb volume showed a significant positive correlation with olfactory function, even after controlling for confounding variables, which is consistent with prior research^29^. However, this correlation was not observed in patients with COVID-19. There are several hypotheses that could explain this absence of correlation. Firstly, many individuals who experienced anosmia or hyposmia during the acute phase of COVID-19 eventually recovered their olfactory function^5^. This restoration could be due to the rapid regeneration of the supporting cells in the olfactory nerve from stem cells^30^. In addition, there is evidence that SARS-CoV-2 can affect WM and gray matter (GM), even in subjects with mild symptoms without hospitalization, which can impact higher processing in brain regions related to the olfactory system^31–33^.

Reduced global and local efficiency was observed in COV + group, including the olfactory-related regions, compared to the COV− group indicating potential disruption in brain network connectivity. Consistent with these findings, a longitudinal imaging study using UK Biobank data revealed a significant detrimental effect of SARS-CoV-2, mainly on the limbic and olfactory cortical system, as well as changes in diffusion measures in regions functionally connected to the piriform cortex, olfactory tubercle, and anterior olfactory nucleus. Moreover, in that study, participants infected with SARS-CoV-2 showed a more pronounced reduction of grey matter thickness in the left parahippocampal gyrus and lateral orbitofrontal cortex^31^. In other studies, WM microstructural alterations were also observed in the brain during the subacute, post-acute, and chronic phases of COVID-19^32–34^, with the potential to disrupt brain network connectivity.

Cerebral plasticity after a sensory loss has been well documented in respect of visual and auditory loss, but less is known about the effects of olfactory input loss on the adult brain. Previous research has demonstrated that acquired anosmia alters GM volume or density in olfactory-related areas, such as the piriform cortex and the orbitofrontal cortex, as well as in non-olfactory-related areas, such as the prefrontal cortex. The higher assortativity in the COV + group suggests that the network nodes are more interconnected with nodes that have comparable properties, which may reflect a compensatory mechanism in response to the disruption of brain network connectivity caused by COVID-19. Our study also showed increased structural connectivity in the posterior parietal cortex, involving multisensory areas such as the postcentral gyrus, inferior parietal gyri, and precuneus, in whole-brain analysis. In a functional study, elevated brain connectivity between the orbitofrontal cortex and the visual association cortex and fusiform gyrus in the COVID-19 anosmia group was identified^14^. One could hypothesize that the absence of olfactory input to these parietal multisensory areas alters the neuronal constellation and promotes a more efficient multisensory-based integration of visual and auditory perception in anosmic individuals^35^.

A recent study on patients with olfactory dysfunction following COVID-19 releaved an increased functional intranetwork connectivity within the default mode network, as well as greater internetwork connectivity between the olfactory and default mode networks. This suggests that the compensatory mechanism of greater intranetwork functional connectivity may help to address the deficits in olfactory processing and overall well-being in COVID-19 patients^36^. In another study, structural and functional connectivity metrics were significantly increased in individuals previously infected with SARS-CoV-2. Greater residual olfactory impairment was associated with more segregated processing in regions functionally connected to the anterior piriform cortex^37^. Similarly, we evidenced an increase in segregation in the orbitofrontal cortex and identified a negative correlation between clustering of whole-brain WM and the olfactory test in the COVID-19 group, which was not observed in the control group.

The present study has some limitations that should be acknowledged. Firstly, this was a cross-sectional study with a non-probabilistic sample, which may limit the generalizability of the findings. Additionally, patients were evaluated only once during the post-acute phase, and there was no serial evaluation at different time points, precluding inferences about the temporal dynamics of abnormalities in the olfactory bulbs and WM. Furthermore, the diffusion parameters selected (e.g., low b-values) may have limited the quantitative diffusion analysis, although they better reflect the clinical protocol context.

In summary, our study highlights the presence of reduced olfactory bulb volume and WM structural network disruption in patients with persistent hyposmia after COVID-19. Our findings suggest that compensatory mechanisms in the parietal sensory and olfactory-related areas may help alleviate the deficiency in olfactory processing in COVID-19 patients. While larger brain connectivity studies are needed to confirm these observations, longitudinal analyses are particularly important to assess the long-term neurological consequences of COVID-19. Further research is also required to explore the potential impact of olfactory dysfunction on quality of life and daily functioning.

## Methods

### Participants

This cross-sectional prospective analytical study was conducted as part of the NeuroCOVID-19 Brazilian Registry (NeuroCovBr, https://www.neurocovbr.com/)^38^, between October 2020 and May 2021 in Brasilia, Brazil. Participants were recruited with a non-probabilistic sampling strategy from a population of health professionals and patients assisted at the Brasilia University Hospital before the implementation of mass vaccination campaigns. We consecutively contacted a list of 364 patients diagnosed with COVID-19 by real-time quantitative reverse transcription polymerase chain reaction (qRT-PCR) to invite them to the study.

The inclusion criteria for the COVID-19 group (COV +) were: (a) diagnosis of SARS-CoV-2 infection confirmed by detection of viral RNA by qRT-PCR testing of a nasopharyngeal swab, without requiring hospitalization during infection, (b) COVID-19-related persistent subjective hyposmia, and (c) age between 18 and 60 years old. Patients were evaluated at least four weeks after the diagnosis of COVID-19 to ensure that the acute phase had already passed. The control group (COV-) was recruited from the same population (patients or health professionals from Brasilia University Hospital) using convenience sampling, with age, sex, and education level matched to the COV + group. Subjects in the COV− group had not been previously infected with SARS-CoV-2, had a negative SARS-CoV IgG/IgM test, and had no olfactory dysfunction.

The exclusion criteria for both groups included (a) pre-existing brain structural disorders (e.g., stroke, epilepsy, multiple sclerosis, neoplasia, hydrocephalus, traumatic brain injury, Parkinson's disease, and dementia), (b) severe psychiatric diseases, (c) MoCA global score of less than 15^39^, (d) MRI contraindications, and (e) illiteracy.

This study was approved by the local ethics committee at the University of Brasilia and adhered to current regulations, such as the Helsinki Declaration. All participants provided written informed consent and underwent clinical, cognitive, and MRI examinations during the same visit.

### Clinical assessment

Demographic and clinical data were collected using electronic forms, including evaluation of neurological, chemosensory, respiratory, and constitutional symptoms. In addition, demographic variables such as age, education, sex, and a list of self-reported comorbidities were obtained.

The Sniffin' Sticks smell identification test (SS-16) was used to evaluate participants' ability to identify odors. This psychophysical test was developed by Burghardt^®^ (Wedel, Germany) and previously adapted to Brazilian Portuguese^26,27^. The test comprises 16 pens containing common and recognizable odorants. The length of each pen is 14 cm (approximately 5.51 in), with an internal diameter of 1.3 cm (about 0.51 in) and a 4 mL cap containing odorless or odorous liquids dissolved in propylene glycol. The participant had to identify the odor using a four-option forced-choice paradigm.

All participants also responded to a cognitive test, MoCA, to screen for cognitive impairment^39,40^. It is a brief 30-point test that assesses attention, executive functions, memory, language, visuoconstructional skills, conceptual thinking, and calculations.

### MRI data acquisition

The MRI was performed using a Philips Achieva 3 T scanner (Best, Netherlands) equipped with an 8-channel SENSE coil. The following MRI sequences were obtained: (1) Three dimensional (3D) T1-weighted sequence, turbo field echo (TFE), sagittal, with field of view (FOV) = 208 × 240 × 256 mm, reconstructed resolution of 1 × 1 × 1 mm, echo time (TE) = min full echo, repetition time (TR) = 2300 ms, TI = 900 ms, two times accelerated acquisition; (2) Diffusion-weighted sequence, axial, with FOV 232 × 232 × 160 mm, reconstructed resolution of 2 × 2 × 2 mm, TE = 71 ms; TR = 3300 ms, 32 directions (b = 800 s/mm^2^); (3) Diffusion-weighted sequence, axial, with FOV 232 × 232 × 160 mm, reconstructed resolution of 2 × 2 × 2 mm, TE = 71 ms; TR = 3300 ms (reversed phase encoded b0); (4) 3D-fluid attenuated inversion recovery (FLAIR) sequence, sagittal, with FOV 256 × 256 × 160 mm, reconstructed resolution of 1.2 × 1 × 1 mm, TE = 119 ms, TR = 4800 ms, TI = 1650 ms. (5) T2-weighted sequence, coronal, with FOV 264 × 204 mm, reconstructed resolution of 0.25 × 0.25 × 1.5 mm, TR = 2500 ms, TE = 80 ms; flip angle = 90, with coverage of the anterior cranial fossa.

### Manual segmentation of the olfactory bulbs

**Figure 3:**
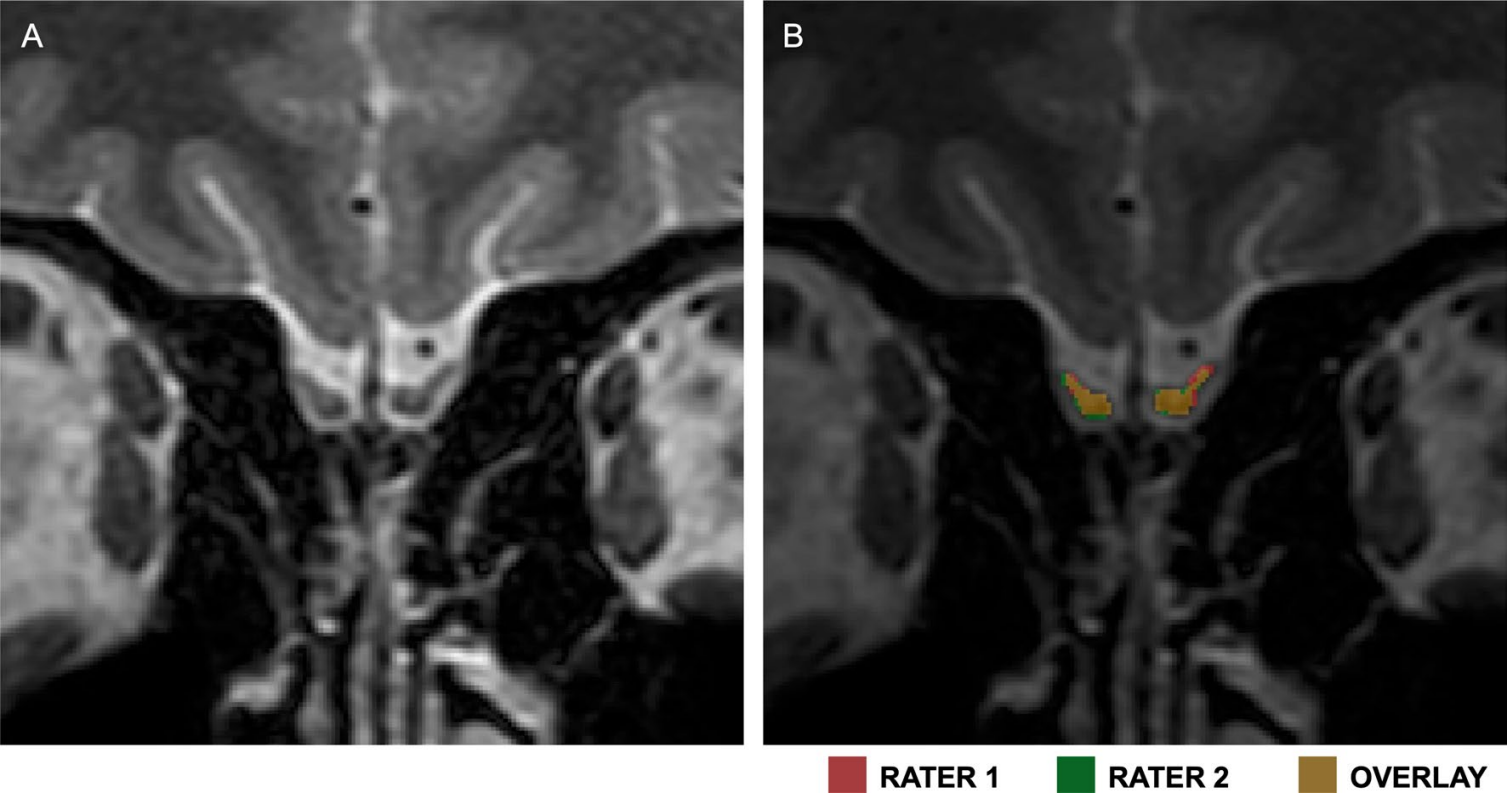
Manual segmentation of the olfactory bulbs. A. Coronal T2-weighted image of a participant. B. Coronal T2-weighted image with segmentations made by the two evaluators. Red: Rater 1; Green: Rater 2; Yellow: Overlap.

Two independent evaluators blinded to clinical and olfactory data manually segmented the volumes of the olfactory bulbs using ITK-SNAP (version 3.8) (Fig. 3)^41^. The limits of the olfactory bulb in the coronal plane were determined by the surrounding cerebrospinal fluid, while an abrupt diameter change defined the posterior boundary of the olfactory bulb at the transition with the olfactory tract^42^. The volumetric measures of the right and left olfactory bulbs were taken independently and then summed. The mean values established by the two evaluators were used in all subsequent analyses. Interobserver agreement was evaluated using Pearson's correlation coefficient and the DSC (Fig. 3).

The estimated total intracranial volume (eTIV) was computed using FreeSurfer (version 7.1.1, http://surfer.nmr.mgh.harvard.edu) which normalized the volumes of the olfactory bulbs to eliminate biases caused by unequal head sizes.

### Diffusion magnetic resonance imaging processing

**Figure 4:**
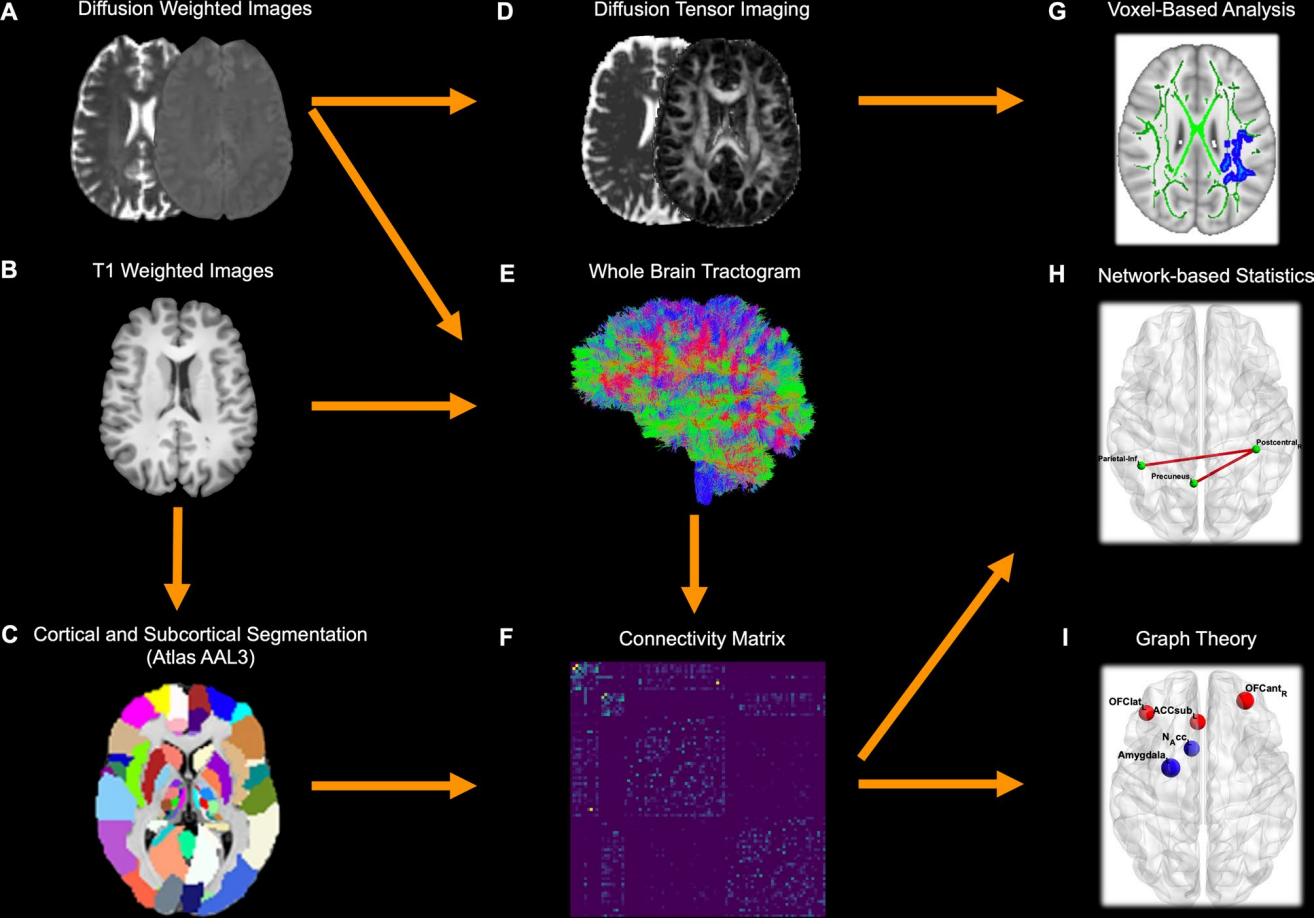
Processing flowchart. ((**A**) and (**B**)). The TractoFlow pipeline processes diffusion-weighted and T1-weighted images (**C**) T1-weighted images are labeled in 171 brain regions of the AAL3 atlas. (**D**) Diffusion MRI-derived measures are computed. (**E**) Whole brain probabilistic tractography is performed using an anatomically constrained particle filter algorithm. (**F**) Extraction of the COMMIT2-weighted connectivity matrix. (**G**) Voxel-based analysis investigated FA, MD, RD, and AD metrics. (**H**) Network-based statistics analysis. (**I**) Graph theory analysis: global and local network. *AAL* automated anatomical labeling, *COMMIT* convex optimization modeling for microstructure informed tractography, *FA* fractional anisotropy, *MD* mean diffusivity, *RD* radial diffusivity, *AD* axial diffusivity.

TractoFlow was used to analyze dMRI and T1-weighted images (Fig. 4)^43^. As an automated tool for processing dMRI, it extracts diffusion tensor imaging (DTI) measures. FA, MD, RD, and AD were calculated. Probabilistic whole-brain anatomically constrained particle filtering tractography was performed on a fiber orientation distribution function (fODF) of maximum spherical harmonics order of 6^44^. The output of TractoFlow was then further processed via advanced steps to generate structural connectomes using SCILPY library version 1.0.0^45^. Then, COMMIT2 with ball & sticks forward model was used to filter the raw tractogram and compute the COMMIT2 weights of each streamline^16,17^.

### Voxel-based diffusion imaging analysis

The TBSS pipeline in FSL (version 6.0)^30^ was used to compare MRI metric differences between the COV + and COV− groups (Fig. 4). The FA maps were nonlinearly aligned to the FMRIB-58 map in the template space of the Montreal Neuroimaging Institute (MNI). The FA skeleton mean was computed following the deformable registration. The FA maps deformation fields were utilized for FA, MD, RD, and AD. The registered maps were projected onto the FA skeleton.

### Network construction

The brain network is composed of nodes and edges. To determine the nodes within the network, we selected 171 grey matter regions of the brain from the AAL3 atlas^46^. Each AAL3 region in standard MNI space was back-transformed to the participant’s native diffusion space. The COMMIT2-weighted tractogram and AAL3 parcellations were used to derive COMMIT2-weighted structural connectivity matrices (Fig. 4). The COMMIT2 weight of a streamline is a measure that quantifies the contribution to the diffusion MRI signal of each streamline and is proportional to the cross-sectional area of the biological fibers along their path. By its turn, the COMMIT2 weight of a connection corresponds to the sum of the individual weights assigned by COMMIT2 to each streamline connecting two parcels of the matrix and was used as a marker of connectivity strength. Through its ability to take into account the tracking bias related to variations in bundle width, the COMMIT2 weight constitutes a more biological proxy than the frequently used streamline count^15^. The possibility to inject priors about brain anatomy and its organization, and not only about microstructural properties, represents a powerful and novel way to tackle the false-positive problem in tractography and brain structural connectivity^16,17^. COMMIT2-weighted 171 × 171 whole-brain matrices were computed.

Three-dimensional projections of structural connections and nodes were visualized using BrainNet Viewer (version 1.42)^47^, for comparison of COMMIT2 weight matrices and graph theory analyses.

### Network‐based statistics

NBS was performed following Zalesky's methods with NBS Connectome (version 1.2) to determine the different connections^48,49^. NBS is a statistical method based on graph theory and is often used to explore differences in the structural connectivity in the brain WM network. Typically, NBS analysis is conducted to identify subnetworks consisting of pairs of nodes and connections whose structural connectivity strength varies significantly between groups.

### Network measures

The Brain Connectivity Toolbox (BCT) computed network measures for each subject^50^. For global networks, betweenness centrality (corresponding to the fraction of all shortest paths in the network), modularity (reflecting the segregation of the network), assortativity (reflecting whether nodes tend to be connected to other nodes with similar strengths), participation (measure of diversity of intermodular connections), clustering coefficient (fraction of connected triangles around a node), mean strength (corresponding to the average of all the nodal strengths, where the nodal strength is the sum of the weights of links connected to the node), global efficiency (corresponding to the average inverse shortest path length in the network and inversely related to the characteristic path length), density (corresponding to the fraction of present connections to possible connections), characteristic path length (average of the shortest path length across all nodes), edge count, and small-worldness (ratio of average clustering coefficient to characteristic path length) were analyzed.

We analyzed regional network measures, calculated for each node, including betweenness centrality (number of shortest paths that pass through a node), clustering (fraction of connected triangles around a node), edge count, local efficiency (average of the inverse shortest path length in the neighborhood a node), nodal strength (sum of weights of links connected to the node), path length (shortest path length across the (average of the shortest path length across all nodes), and participation (a measure of the diversity of intermodular connections of a node).

Local network measures were calculated for the olfactory-related brain regions (olfactory cortex, gyrus rectus, medial orbital gyrus, anterior orbital gyrus, posterior orbital gyrus, lateral orbital gyrus, insula, hippocampus, parahippocampal gyrus, amygdala, caudate nucleus, putamen, pallidum, thalamus [mediodorsal medial nucleus and mediodorsal lateral nucleus], anterior cingulate cortex [subgenual, pregenual and supracallosal], and nucleus accumbens)^51,52^.

### MRI quality control

The MRI images were inspected for significant gross geometric distortion, mass movement, and signal drop artifacts to ensure their quality. For T1-weighted and dMRI images, a Nextflow pipeline for dMRI quality control (Dmriqc-flow) was also utilized^53^.

### Statistical analysis

#### Demographic and clinical assessments

The demographic and clinical characteristics of the groups were compared using independent-sample t-tests for normally distributed continuous variables, Mann–Whitney tests for nonnormally distributed continuous variables, and χ^2^ for categorical variables. Fulfillment of the normality assumption was inspected through visual examination of variable distributions and the Shapiro–Wilk test. The significance level was set at p < 0.05. All statistical analyses were performed in R, version 4.1.0 (*R Foundation for Statistical Computing*, Vienna, Austria).

#### Segmentation of the olfactory bulbs

The olfactory bulb volumes were compared using a t-test for independent samples. To account for multiple comparisons, the results were adjusted using the False Discovery Rate (FDR) method^54^. The eTIV corrected the volumes obtained with the formula: (volume of the olfactory bulb/eTIV) × 100.

An analysis of covariance (ANCOVA) was performed to compare the normalized total olfactory bulb volume between groups, while controlling for variables such as sex, age, and allergic rhinosinusitis. As necessary, significant p-values were adjusted using post hoc Bonferroni tests (p < 0.05).

The level of interobserver agreement for the segmentation of the olfactory bulbs was assessed by the Pearson's correlation coefficient and the DSC. The DSC is an overlap similarity index that reflects agreement in size and location. It ranges from 0 (no overlap) to 1 (complete overlap) (Fig. 3). A satisfactory overlap exists when DSC > 0.70^55^.

#### Voxel-based diffusion imaging analysis

To test for group differences, a general linear model (GLM) with contrast was performed on VBA data. The TBSS framework^30^ includes nonparametric permutation testing (5000 permutations) to correct multiple comparisons and *threshold-free cluster enhancement* (TFCE). Age and sex were used as nuisance covariates. Results were considered significant at *p* < 0.05, TFCE corrected for multiple comparisons. WM regions were named according to the Johns Hopkins University white-matter tractography atlas.

#### Network‐based statistics

Between-group differences (COV−  > COV + and COV- < COV + contrasts) were tested on structural connectivity matrices for a range of primary thresholds (from t = 2.5 to t = 3.5), with age and sex as nuisance variables. Five thousand permutations were used, with intensity as the measure of network size and a statistical significance threshold set at p < 0.05.

#### Network metrics

Between-group differences were tested with either Mann–Whitney (modularity, clustering, and nodal strength) or independent-sample t-tests (other global network metrics). A GLM was used to analyze the local network metrics differences in olfactory-related brain areas between the control and COVID-19 groups, using age and sex as covariates. All results were corrected using the FDR method^54^.

We performed a partial correlation analysis between global network measures, SS-16 test score, and normalized total olfactory bulb volume, adjusting for age, sex, education, comorbidities (allergic rhinosinusitis and migraine), and time between COVID-19 diagnosis and study clinical/imaging procedures. Data were analyzed using Spearman's coefficient. Statistical significance was defined as a two-tailed p < 0.05.

### Supplementary Information

Supplementary Information.

## Data Availability

The anonymized dataset that supports these study findings is available upon reasonable request from the corresponding author from a qualified investigator if the intent is to increase reproducibility. The data were not publicly available because of privacy or ethical restrictions.
