# Supplementary material for: Altered structural connectivity in olfactory disfunction after mild COVID-19 using probabilistic tractography
Source: Sci Rep. 2023 Aug 9;13:12886. doi: 10.1038/s41598-023-40115-7 (PMC10412532; doi:10.1038/s41598-023-40115-7)
Supplement: Supplementary file 1 — Supplementary Information. [file 41598_2023_40115_MOESM1_ESM.pdf]

Altered structural connectivity in olfactory disfunction after mild COVID-19 using probabilistic tractography

Authors:
Diógenes Diego de Carvalho Bispo[1,2,3,*#], Pedro Renato de Paula Brandão[4,5,#], Danilo Assis Pereira[6], Fernando Bisinoto Maluf[3], Bruna Arrais Dias[3], Hugo Rafael Paranhos[3], Felipe von Glehn[2,5], Augusto César Penalva de Oliveira[7], Alexandre Anderson de Sousa Munhoz Soares[2], Maxime Descoteaux[8,9,##], and Neysa Aparecida Tinoco Regattieri[2,##]
[#] Both authors share the first authorship.
[##] Both authors share senior authorship.

Supplementary Information

Supplementary Table S1. Group comparison of COMMIT2-weighted connectivity matrices of the whole-brain network between COV- and COV+. Significance was thresholded at $p < 0.05$. Permutations = 5,000.

CONTROL > COVID-19			
Node 1	Node 2	t	$t_{thr} = 3.0$
Postcentral gyrus R	Inferior parietal gyrus L	3.60	X
Postcentral gyrus R	Precuneus L	3.83	X

Abbreviations: L, left; R, right; thr, threshold.

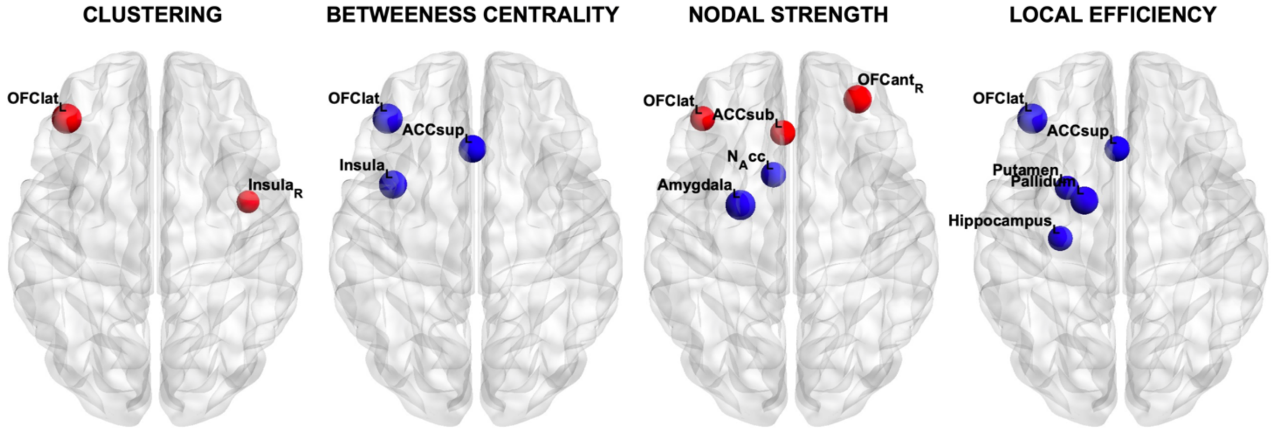

Supplementary Figure S1. Difference of local network metrics in olfactory-related regions between COV+ and COV- ($p < 0.05$ uncorrected). The size of the node represents the significance of difference between two groups.
Abbreviations: OFClat, lateral orbital gyrus; OFCant, anterior orbital gyrus; ACCsup, anterior cingulate cortex (supracallosal); ACCsub, anterior cingulate cortex (subgenual); NAcc, nucleus accumbens, R, right; L, left.
